# Synthesis and Biological Evaluation of New Pyridone-Annelated Isoindigos as Anti-Proliferative Agents

**DOI:** 10.3390/molecules190913076

**Published:** 2014-08-25

**Authors:** Ayman M. Saleh, Randa M. Al-As’ad, Mustafa M. El-Abadelah, Salim S. Sabri, Jalal A. Zahra, Ahmed S. Alaskar, Ahmad Aljada

**Affiliations:** 1 Department of Basic Medical Sciences, College of Medicine, King Saud Bin Abdulaziz University for Health Sciences (KSAU-HS), P.O. Box 3660, Riyadh 11481, Kingdom of Saudi Arabia; E-Mail: aljadaa@ksau-hs.edu.sa; 2 King Abdullah International Medical Research Center (KAIMRC), National Guard Health Affairs, P.O. Box 22490, Riyadh 11426, Kingdom of Saudi Arabia; E-Mails: askaras@ngha.med.sa (A.S.A.); aljadaa@ngha.med.sa (A.A.); 3 Chemistry Department, Faculty of Science, The University of Jordan, Amman 11942, Jordan; E-Mails: randaalasad@yahoo.com (R.M.A.-A.); s.sabri@ju.edu.jo (S.S.S.); zahra@ju.edu.jo (J.A.Z.)

**Keywords:** pyridone-annelated isoindigos, 5'-halogeno derivatives, isoindigo, anticancer compounds, antiproliferative activity, apoptosis, K562 cells, THP-1 cells, HepG2 cells, MCF-7 cells, Caco-2 cells, HEK-293 cells, L-929 cells, MTT assay

## Abstract

A selected set of substituted pyridone-annelated isoindigos **3a**–**f** has been synthesized via interaction of 5- and 6-substituted oxindoles **2a**–**f** with 6-ethyl-1,2,9-trioxopyrrolo[3,2-*f*]quinoline-8-carboxylic acid (**1**) in acetic acid at reflux. Among these isoindigos, the 5'-chloro and 5'-bromo derivatives **3b** and **3d** show strong and selective antiproliferative activities against a panel of human hematological and solid tumor cell-lines, but not against noncancerous cells, suggesting their potential use as anticancer agents. In all the tested cell lines, compound **3b** was a 25%–50% more potent inhibitor of cell growth than **3d**, suggesting the critical role of the substitution at 5'**-**position of the benzo-ring E. The IC_50_ values after 48 hours incubation with the 5'-chloro compound **3b** were 6.60 µM in K562, 8.21 µM in THP-1, 8.97 µM in HepG2, 11.94 µM in MCF-7 and 14.59 µM in Caco-2 cancer cells, while the IC_50_ values in noncancerous HEK-293 and L-929 were 30.65 µM and 40.40 µM, respectively. In addition, compound **3b** induced higher levels apoptosis in K562 cells than **3d**, as determined by annexin V/7-AAD flowcytometry analysis. Therefore, further characterization of the antitproliferative mechanisms of compounds **3b** and **3d** may provide a novel chemotherapeutic agents.

## 1. Introduction

Leukemia is one of the most fatal hematological cancers worldwide. Human chronic myelocytic leukemia (CML), a highly common type of leukemia, is a myeloproliferative disorder of blood cells that is characterized by increased proliferation of undifferentiated granulocytic progenitors. CML is induced by constitutive expression and activation of the fusion gene Bcr-Abl encoding a tyrosine kinase, which develops from a translocation between chromosomes 9 and 22 to generate Philadelphia chromosome [1]. Imatinib mesylate (Glivec^®^), a specific inhibitor of Bcr-Abl activity, was developed as the first anticancer molecule to treat CML patients. However, a high percentage of CML patients were reported to develop resistance to Glivec^®^ as a result of increased mutation incidents in the Abl kinase domain, indicating the requirement for new treatment regimen [1,2].

**Figure 1:**
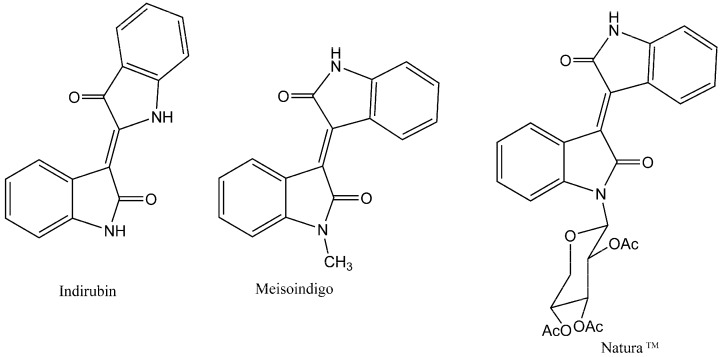
Structures of indurbin, meisoindigo and Natura^TM^.

In the course of our laboratory studies aiming to synthesize compounds with potential antiproliferative activities towards leukemia cells, we have initiated a chemical synthesis program of indigoid derivatives. Indigoids are a class of *bis*-indole alkaloids, obtained from *Baphicacanthus cusia* (Nees) Bremek. (Acanthaceae), *Indigofera suffruticosa* Mill. (Fabaceae), *Indigofera tinctoria* L. (Fabaceae), *Isatis tinctoria* L. (Brassicaceae), and *Polygonum tinctorium* Ait. (Polygonaceae) [3,4]. There are three isomeric forms of the bisindoles—indigo, indirubin and isoindigo. While the colored indigo is mainly used as a textile dye, indirubin (Figure 1) was identified as the active ingredient of a traditional Chinese recipe (Danggui Longhui Wan) that was used for the treatment of chronic myelogenous leukemia (CML) [5,6]. Indirubin exerts its antileukemic effect by competing with ATP for binding to the catalytic subunit of cyclin-dependent kinases (CDKs), leading to the inhibition of these enzymes [4,7,8]. Meisoindigo, (1-methylisoindigo, Figure 1), was developed to improve the solubility in water and consequently override the antileukemic properties of indirubin. This compound showed significant activities against cancer cells through multi-signaling pathways including inhibition of DNA biosynthesis and assembly of microtubules, arresting leukemia cells at G1 phase of the cell cycle, induction of cell differentiation and maturation leading to complete inhibition of cell growth without a decrease in cell viability, and down-regulation of c-myb gene expression [6,9,10,11]. Meisoindigo has been subjected to clinical trials [6,10,11,12], and is used as an indirubin substitute in the People’s Republic of China for the treatment of CML [13].

In association with the design of meisoindigo analogs with increased bioavailability and bioactivity, the isoindigo Natura^TM^ (1-(β-d-triacetylxylopyranosyl) isoindigo, Figure 1), was synthesized [14,15] and showed antiproliferative activities that only slightly exceed those of meisoindigo in different cancer cell lines [14,15,16]. Like meisoindigo, Natura^TM^ induced apoptosis, inhibited CDKs and effectively arrested tumor growth in mice transplanted with Walker 256 cancer cells [14,15,16]. However, Natura^TM^ is almost completely insoluble in water, a property which hinders its applications in cancer chemotherapy.

**Scheme 1:**
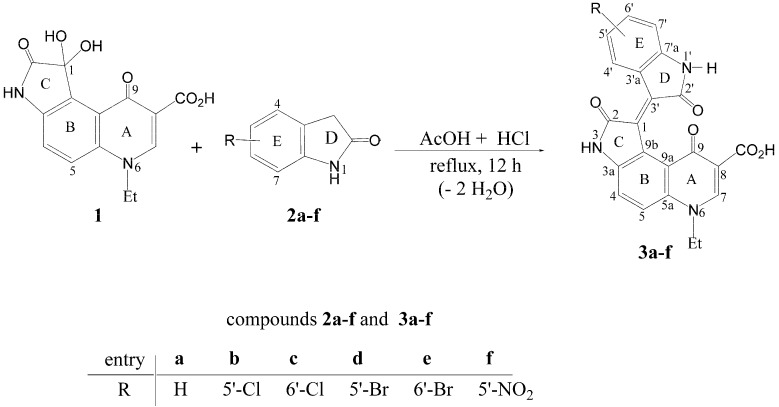
Synthesis of the pyridone-annelated isoindigos **3a**–**f**.

While these compounds have shown some promising pharmaceutical efficacy, there is still a need for additional compounds that are effective for treating CML patients. Accordingly, and in line with developing new isoindigos, we thought it was worthwhile to prepare some pyridone-annelated isoindigos to evaluate their antitumor activities. Herein, we report on the synthesis of new pyridone-annelated isoindigo derivatives, namely the 6-ethyl-2,9-dioxo-1-(2'-oxoindolin-3'-ylidene)-2,3,6,9-tetrahydro-1*H*-pyrrolo[3,2-*f*]quinoline-8-carboxylic acids **3a**–**f** using the acid-catalyzed reactions of 6-ethyl-1,2,9-trioxopyrrolo[3,2-*f*]quinoline-8-carboxylic acid (**1**) [17] with oxindoles **2a**–**f** (Scheme 1), and the evaluation of their antiproliferative activities against the human chronic myelogenous leukemia K562 and other hematological and solid tumor cell lines. We show that two of our synthesized compounds having a chlorine and bromine substitution at the 5'-position of the benzo-ring E, effectively inhibit proliferation of K562 cells in a dose- and time-dependent fashion with IC_50_ values ranging from 4 to 19 µM, depending on the exposure time to the compound. In addition to K562 cells, both compounds effectively inhibit the growth of other hematological cancer cells (human acute monocytic leukemia THP-1), and the human solid tumor derived hepatocellular carcinoma (HepG2), breast adenocarcinoma cancer (MCF-7) and colorectal adenocarcinoma cells (Caco-2) at low doses of either compound. However, much higher concentrations of these compounds are required to inhibit the growth of noncancerous human epithelial cells HEK-293 or mouse cutaneous derived fibroblast. Our results show that both compounds **3b** and **3d** effectively induce apoptosis in K562 leukemic cells. Therefore, these novel isoindigo molecules are promising candidates for further investigation of their antiproliferative mechanisms against different tumors.

## 2. Results and Discussion

### 2.1. Chemistry

The key intermediate in this synthesis is 6-ethyl-1,2,9-trioxopyrrolo[3,2-*f*]quinoline-8-carboxylic acid **(1**), the preparation of which has been recently reported [17]. The targeted isoindigos **3a**–**f** were synthesized by acid-catalyzed crossed aldol–condensation reaction between 6-ethyl-1,2,9-trioxopyrrolo[3,2-*f*]quinoline-8-carboxylic acid (**1**) [17] and the appropriate oxindoles **2a**–**f** under reflux (Scheme 1). The new compounds **3a**–**f** were characterized by elemental analyses, MS and NMR spectral data. These data, detailed in the Experimental section, are in conformity with the assigned structures. Thus, the mass spectra displayed the correct molecular ion peaks for which the measured high-resolution mass spectral (HRMS) data were in good agreement with the calculated values. DEPT and 2D (COSY, HMQS, HMBC) experiments showed correlations that helped in the ^1^H and ^13^C signal assignments to the different carbons and their attached, and/or neighboring hydrogens. In HMBC experiments, distinct long-range “three-bond” correlations were observed between 4-H and each of C-9b and C-5a, between 5-H and each of C-3a and C-9a, between 7-H and each of C-5a, C-9 and CO_2_H, as well as between H-N(3) and each of C-1 and C-9b, while H-N(1') was correlated with C-3' and C-3'a. In compounds **3b**, **3d** and **3f**, “three-bond” correlations were also observed between 7'-H and each of C-5' and C-3'a; likewise in compounds **3c** and **3e**, 4'-H was correlated with C-6' and C-7'a.

### 2.2. In Vitro Antiproliferative Activity of the Synthesized Isoindigoid Derivatives ***3a**–**f***

**Figure 2:**
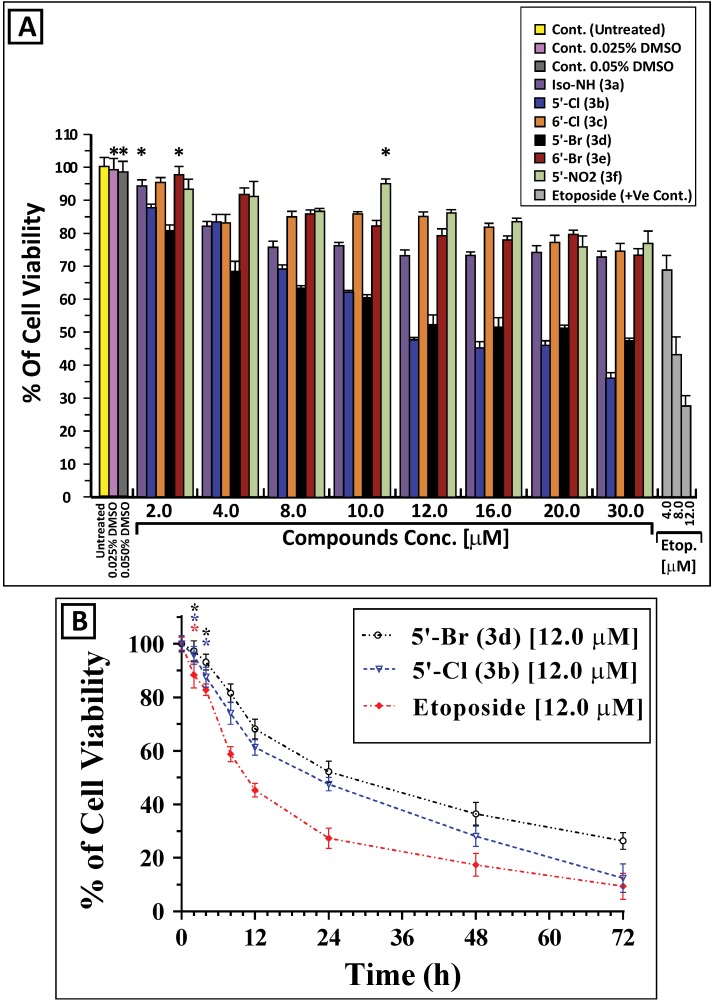
The isoindigo compounds **3b** and **3d** inhibit the growth of K562 cells in a dose- and time-dependent manner. (**A**) K562 cells were treated with varying concentrations of compounds **3a** to **3f** (0.0 to 30.0 µM) for 24 h and the antiproliferative activity was analyzed by MTT assay as described in the methods. (**B**) K562 cells were incubated with 12.0 µM of either compound **3b** or **3d** for different periods of time (0 to 72 h) and cell viability was assessed by the MTT assay. The controls (in the presence or absence of the solvent vehicle DMSO) represent cells incubated under similar conditions in the absence of the test compound. The percentage of cell viability in each sample was expressed relative to untreated control, which was considered as a 100%. K562 cells were also treated with 4.0, 8.0 and 12.0 µM etoposide under similar conditions as positive controls. The results shown represent the mean ± SD of three independent trials. Statistical analysis showed that all samples are significantly different (*p* < 0.05), except *-marked values which are statistically insignificant (*p* < 0.01) when compared to untreated control without the test compound or etoposide.

**Figure 3:**
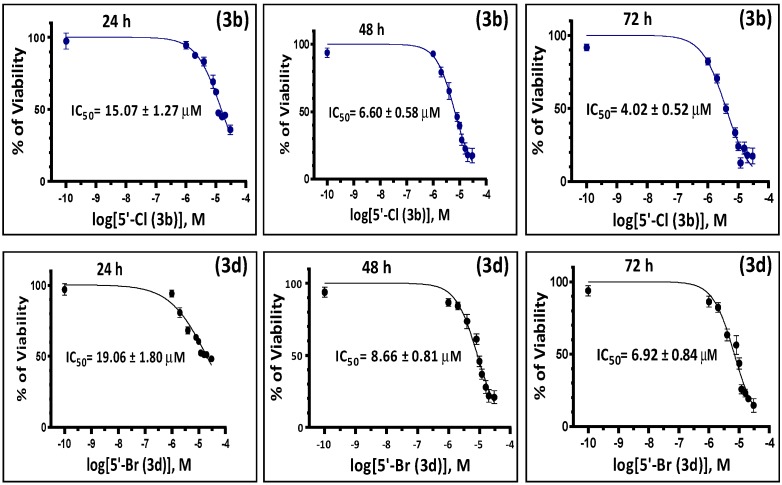
The IC_50_ values of compounds **3b** and **3d** determined in K562 cells at different time points. K562 cells were treated with varying concentrations of compounds **3b** or **3d** (0.0 to 40.0 µM) for different periods of time (24, 48 and 72 h) and their antiproliferative activity was analyzed by MTT viability assay. The sigmoidal curves shown were obtained by plotting the percentages of viability (mean ± SD of three independent experiments, three replicates for each concentration in every experiment were used) *versus* logarithmic molar concentrations of the test compounds at the three different time points, and the IC_50_ value was determined using a non-linear regression analysis of GraphPad Prism 6 software.

The antiproliferative activities of the synthesized molecules were initially evaluated in human chronic lymphocytic leukemia cells K562. The effect of the six derivatives on the viability of K562 was assessed by an MTT assay [18,19] as described in the methods. In all MTT tests performed, the known antiproliferative agent etoposide [20] was used as a positive control to validate the accuracy of the assay. Under the conditions used, only two of the tested compounds (compounds **3b** and **3d**) have shown a significant dose-dependent cytotoxicity to K562 cells (Figure 2A). Compounds **3b** and **3d** also inhibited the growth of K562 cells in a time-dependent fashion (Figure 2B). Interestingly, compound **3b** showed 25%–50% more cytotoxic effect than **3d** in all the tested doses and time points, and was reflected by the IC_50_ of these compounds when incubated with cultured K562 cells. The IC_50_ values of compounds **3b**/**3d** in K562 cells were 15.07 ± 1.27/19.06 ± 1.80, 6.60 ± 0.58/8.66 ± 0.81 and 4.02 ± 0.52/6.92 ± 0.84 µM after 24, 48 and 72 h (Figure 3), respectively, which suggest the importance of chlorine substitution at the 5'**-**position of the benzo-ring E.

Based on the above results, the most potent cytotoxic compound towards K562 cells is **3b**, followed by **3d**. This cytotoxicity is probably due to the presence of chlorine and bromine atoms in the 5'**-**position of the benzo-ring E, because having either ion substitution at the 6'**-**position (compounds **3c** and **3e**) significantly abolished the cytotoxicity of the molecules. Furthermore, the lack of either chlorine or bromine atom on the benzo-ring E (compound **3a**) or their substitution with nitro group at the same 5'**-**position (compound **3f**) significantly inhibited their antiproliferative activities when compared to **3b** and **3d**. This indicates the critical rule of structure-activity relationship (SAR) of these molecules. These results are in agreement with related studies that show the strong correlations between halogen substitution patterns, at 5', 6', and 7' positions of the aromatic ring of isatins [21] and phenyl group of isoindigos [22], and the antiproliferative activities against cancer cells.

**Figure 4:**
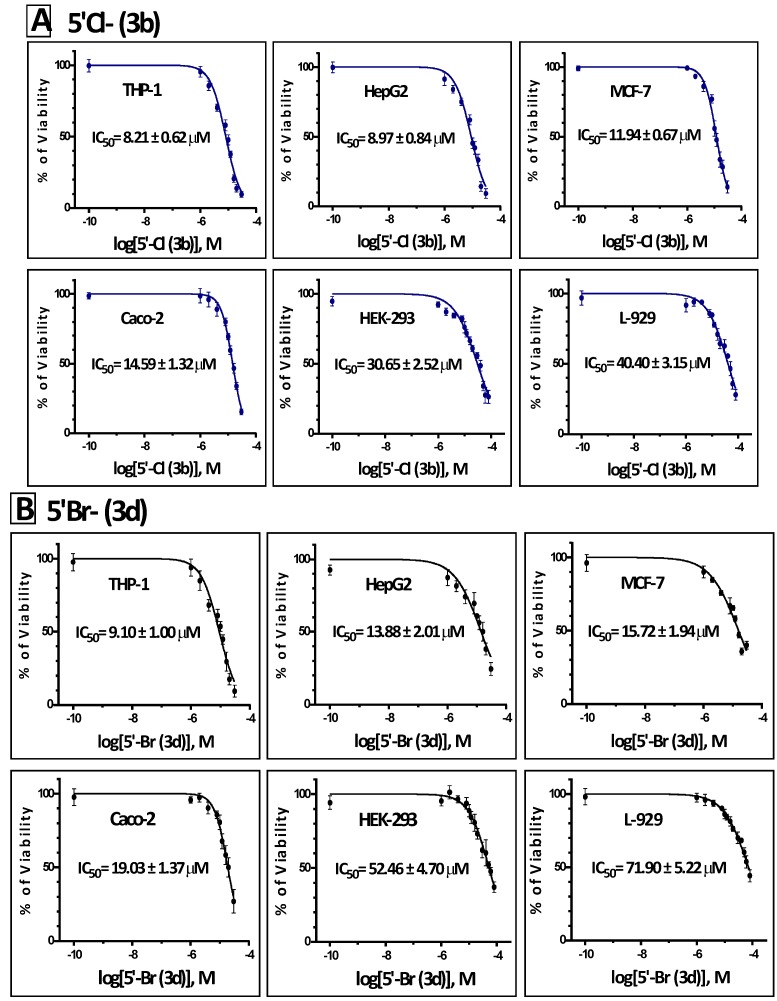
Compounds **3b** and **3d** inhibit the growth of various hematological and solid tumor cell lines but not noncancerous cells at low IC_50_ values. The IC_50_ values of compounds **3b** (**A**) and **3d** (**B**) were determined for cultured cancer cells THP-1, HepG2, MCF-7 and Caco-2 and noncancerous HEK-293 and L-929 after incubation for 48 h with varying concentrations of the test compound as described in Figure 2 using the same GraphPad Prism 6 software.

**Figure 5:**
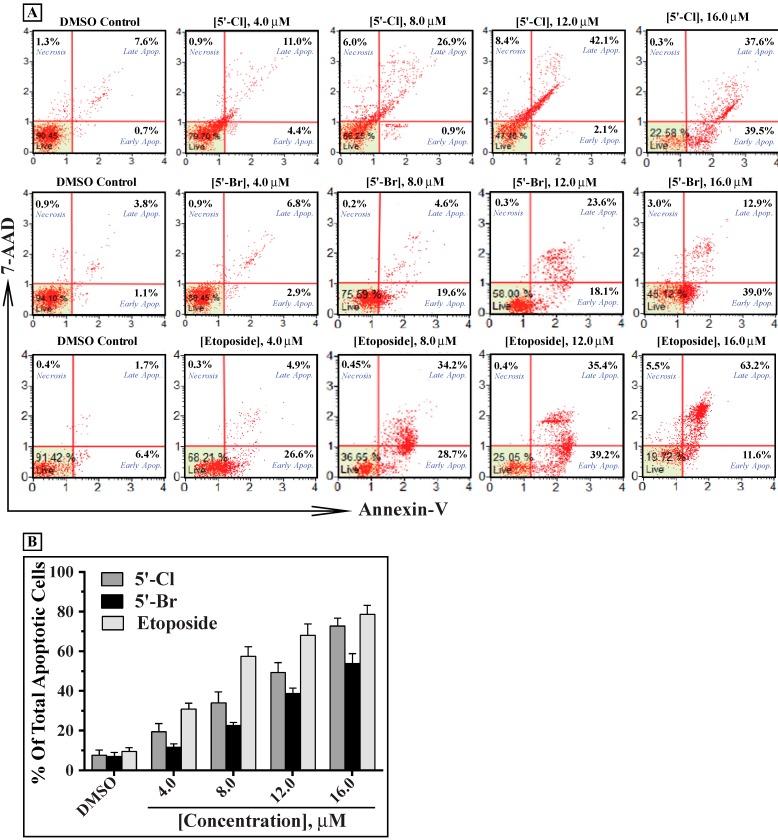
Compounds **3b** and **3d** induce apoptosis in K562 cells in a dose-dependent manner. Cultured K562 cells were treated with varying concentration of compounds **3b**, **3d** or etoposide (0.0 to 16.0 μM) for 24 h and apoptosis was analyzed by a flow cytometer after staining with FITC-annexin-V and 7-AAD as described in the experimental section. The scattered blots (**A**) showing the percentages of early (Annexin^+^, 7-AAD^−^) and late apoptosis (Annexin^+^, 7-AAD^+^), and necrosis (Annexin, 7-AAD^+^) are representation of one experiment. The graph (**B**) represents the mean percentages ± SD of total apoptosis (early and late apoptosis) of three independent experiments for each concentration of the test compound. K562 cells were treated with different concentrations of etoposide as positive controls. Statistical analysis showed that all samples are significantly different (*p* < 0.05), when compared to their untreated controls (DMSO).

In addition to the effect on K562 cells, we have also evaluated the potential antiproliferative activities of compounds **3b** and **3d** on different hematological and solid tumor cell lines. Both compounds inhibited the growth of THP-1, HepG2, MCF-7 and Caco-2 cells in a dose dependent manner of **3b** (Figure 4A) and **3d** (Figure 4B). In all the tested cell lines, compound **3b** was more potent inhibitor of growth than **3d**. The IC_50_ values for compounds **3b**/**3d** were 8.21 ± 0.62/9.10 ± 1.00 μM in THP-1, 8.97 ± 0.84/13.88 ± 2.01 μM in HepG2, 11.94 ± 0.67/15.72 ± 1.94 μM in MCF-7 and 14.59 ± 1.32/19.03 ± 1.37 μM in Caco-2 cells (Figure 4). In all these tested cell lines, compound **3b** was a significantly more potent inhibitor of growth than **3d**. Interestingly, the IC_50_ values of compounds **3b**/**3d** in the noncancerous human embryonic kidney epithelial cells HEK-293 and the mouse subcutaneous connective tissue fibroblast L-929 were much higher than any of the above cancer cells (30.65 ± 2.52/52.46 ± 4.70 μM and 40.40 ± 3.15/71.90 ± 5.22 μM, respectively), suggesting the selectivity of these two compounds to inhibit tumor cells (Figure 4). To further characterize the cytotoxicity of compounds **3b** and **3d**, we evaluated their effects on inducing apoptosis of the cultured K562 cells (Figure 5). Our data indicate that both compounds induced apoptosis in a dose-dependent manner. In all the tested concentrations, compound **3b** showed more apoptotic effect than **3d**. The apoptosis data are in agreement with the results of MTT, and suggests that the antiproliferative activity of these two compounds is mediated by apoptosis.

Several reports have shown that the antiproliferative activities of isoindigos towards various cancer cells are due to deregulation of cell cycle and/or induction of apoptosis [6,7,8,9,10,11]. Indirubin molecules from the Chinese herb-Qing Dai, exhibit their anticancer activity through modulating cyclin-dependent kinases (CDKs), which will arrest cell cycle progression leading to cell death by apoptosis [23]. Multiple indirubin and isoindigo derivatives have been synthesized and shown to inhibit cyclin-dependent kinases (CDKs) and glycogen-synthase kinase (GSK-3β) with varying degrees of potency [6,7,8,9,10,11,14,15,16,19]. Recently, a novel 7-azaisoindigo derivative [namely N(1)-(*n*-butyl)-7-azaisoindigo] have been shown to trigger apoptosis through reactive oxygen species (ROS), disfunctioning of the mitochondria and activation of caspases [24]. Comprehensive mechanistic studies aiming to determine the cellular pathways responsible for the antiproliferative activities of compounds **3b** and **3d**, including cell cycle and apoptosis, are currently under investigations in our laboratories.

A large group of diversely substituted isoindigos, mainly at the lactam *N*(1) locus, have been prepared and evaluated for their antiproliferative and antileukemic activities [25,26,27,28]. Isoindigos are generally prepared by reacting the appropriate isatin (1 equiv) and oxindole (1 equiv) in acetic acid at reflux [25,26,27] or using microwave irradiation at 200 °C for 30 min [27]. Other prospective synthetic routes include dimerization of ketocarbenes generated from derivatives of isatin [29,30], as well as interaction of sodium phosphonates with *N*-methylisatoic anhydride or *N*-methylisatin [31,32]. Despite the progress in the synthesis of isoindigo derivatives and assessing their antiproliferative activities, the bioavailability and selectivity of this group of molecules towards tumor cells restrict their pharmacological application in cancer treatment. Therefore, synthesis of novel isoindigoid compounds with prominent selectivity and availability to cancerous cells, and detailed characterization of their antitumor mechanisms remain a challenging and important research field.

## 3. Experimental Section

### 3.1. Chemicals

The chemicals used in this study (diethylethoxymethylene malonate, *p-*nitroaniline, diphenyl ether, iodoethane, chloral hydrate, hydroxylamine hydrochloride, and anhydrous SnCl_2_) were purchased from Acros Organics (Thermo Fisher Scientific, Geel, Belgium) and were used as received. The required oxindoles **2a**–**f** (oxindole, 5-nitrooxindole, 5-chlorooxindole, 6-chlorooxindole, 5-bromooxindole and 6-bromooxindole), 4,5-dimethylthiazol-2yl)-2,5-diphenyltetrazolium bromide (MTT) and etoposide [4'-demethylepipodophyllotoxin 9-(4,6-*O*-ethylidene-β-d-glucopyranoside)] were acquired from Sigma-Aldrich Co. (St Louis, MO, USA). Cell culture media (RPMI 1640, MEM and DMEM), penicillin-streptomycin, and fetal bovine serum (FBS) were purchased from Invitrogen (Carlsbad, CA, USA).

### 3.2. Instrumentation

^1^H and ^13^C-NMR spectra were recorded on a 500 MHz spectrometer (Bruker AVANCE-III, Bruker BioSpin Corporation, Billerica, MA, USA). Chemical shifts are expressed in ppm (δ units), with TMS as internal standard; *J*-values for ^1^H-^1^H coupling constants were given in Hertz. High resolution mass spectra (HRMS) were acquired (in positive or negative mode) using electrospray ion trap (ESI) technique by collision-induced dissociation on a Bruker APEX-4 (7-Tesla) instrument. The samples were dissolved in acetonitrile, diluted in spray solution (methanol/water 1:1 v/v + 0.1% formic acid) and infused using a syringe pump at a flow rate of 2 µL/min. External calibration was conducted using arginine cluster in a mass range *m/z* 175–871. IR spectra were recorded on a TENSOR 27 FT-IR with ART unit from Bruker. Elemental analyses were performed on a Euro Vector elemental analyzer, model EA 3000.

### 3.3. Synthesis of 6-Ethyl-2,9-dioxo-2,3,6,9-tetrahydro-1H-pyrrolo[3,2-f]quinoline-8-carboxylic Acid (**1**)

The title compound has been prepared in two-steps starting with ethyl 6-amino-1-ethyl-4-oxo-1,4-dihydroquinoline-3-carboxylate according to the method described by Sandmeyer [33,34], and following the published procedure [17].

### 3.4. General Procedure for the Synthesis of Pyridone-Annelated Isoindigos ***3a**–**f***

The title compounds were prepared by adopting the general versatile method reported by Papageorgiou and Borer [28]: A suspension of the appropriate oxindole **2a**–**f** (1.0 mmol) and 6-ethyl-2,9-dioxo-2,3,6,9-tetrahydro-1*H*-pyrrolo[3,2-*f*]quinoline-8-carboxylic acid (**1**) (0.3 g, 1.0 mmol) in glacial acetic acid (5 mL) and few drops of concentrated hydrochloric acid was heated overnight under reflux. Thereafter, the reaction mixture was allowed to cool to room temperature. The resulting solid product (the targeted isoindigo) was collected by suction filtration, washed with ethyl acetate (2 × 5 mL) and dried.

*(E)-6-Ethyl-2,9-dioxo-1-(2'-oxoindolin-3'-ylidene)-2,3,6,9-tetrahydro-1H-pyrrolo[3,2-f]quinoline-8-carboxylic acid* (**3a**): Yield: 0.37 g (92%); m.p. > 300 °C. ^1^H-NMR (DMSO-*d*_6_): δ = 1.45 (t, *J* = 7.0 Hz, 3H, CH_3_CH_2_), 4.60 (q, *J* = 7.0, 2H, CH_2_Me), 6.80 (d, *J* = 7.7 Hz, 1H, 7'-H), 7.00 (ddd, *J* = 7.7 Hz, 7.7 Hz, 1.0 Hz, 1H, 5'-H), 7.37 (ddd, *J* = 7.7 Hz, 7.7 Hz, 1.0 Hz, 1H, 6'-H), 7.38 (d, *J* = 9.1 Hz, 1H, 4-H), 7.98 (d, *J* = 9.1 Hz, 1H, 5-H), 8.75 (d, *J* = 7.7 Hz, 1H, 4'-H), 8.99 (s, 1H, 7-H), 10.54 (s, 1H, N(1')-H/exchangeable with D_2_O), 10.99 (s, 1H, N(3)-H/exchangeable with D_2_O), 15.30 (br s, 1H, CO_2_H/exchangeable with D_2_O).^13^C-NMR (DMSO-*d*_6_): δ = 15.5 (CH_3_CH_2_), 50.1 (CH_2_Me), 108.7 (C-8), 110.3 (C-7'), 116.6 (C-4), 117.9 (C-9b), 121.7 (C-5'), 121.9 (C-3'a), 122.7 (C-5), 124.7 (C-9a), 129.3 (C-1), 130.6 (C-4'), 134.2 (C-6'), 134.4 (C-5a), 138.0 (C-3'), 144.6 (C-3a), 145.7 (C-7'a), 147.1 (C-7), 166.9 (CO_2_H), 168.1 (C-2'), 169.8 (C-2), 177.6 (C-9). HMRS ((+)-ESI): *m/z* = 402.10849 (calcd. 402.10845 for C_22_H_16_N_3_O_5_, [M+H]^+^); *m/z* = 424.09050 (calcd. 424.09039 for C_22_H_16_N_3_O_5_Na), [M+Na]^+^. IR: ν 3411, 3228, 2919, 2849, 1709, 1654, 1612, 1533, 1453 cm^−1^. *Anal*. Calcd. for C_22_H_15_N_3_O_5_ (401.37): C, 65.83; H, 3.77; N, 10.47. Found C, 65.62; H, 3.64; N, 10.25.

*(E)-1-(5'-Chloro-2'-oxoindolin-3'-ylidene)-6-ethyl-2,3,6,9-tetrahydro-2,9-dioxo-1H-pyrrolo[3,2-f]quinoline-8-carboxylic acid* (**3b**): Yield: 0.41 g (93%); m.p. > 300 °C.^1^H-NMR (DMSO-*d*_6_): δ = 1.43 (t, *J* = 7.0 Hz, 3H, CH_3_CH_2_), 4.57 (q, *J* = 7.0 Hz, 2H, CH_2_Me), 6.88 (d, *J* = 9.0 Hz, 1H, 7'-H), 7.42 (d, *J* = 8.0 Hz, 1H, 4-H), 7.46 (d, *J* = 9.0 Hz, 1H, 6'-H), 7.92 (d, *J* = 8.0 Hz, 1H, 5-H), 8.94 (s, 1H, 7-H), 9.19 (s, 1H, 4'-H), 10.71 (s, 1H, N(3)-H/exchangeable with D_2_O), 11.05 (s, 1H, N(1')-H/exchangeable with D_2_O), 15.21 (s, 1H, CO_2_H/exchangeable with D_2_O). ^13^C-NMR (DMSO-*d*_6_): δ = 14.9 (CH_3_CH_2_), 49.8 (CH_2_Me), 107.7 (C-8), 111.5 (C-7'), 116.4 (C-4), 118.4 (C-5), 123.1 (C-9a), 123.2 (C-3'a), 124.3 (C-9b), 125.6 (C-5'), 129.4 (C-4'), 132.9 (C-6'), 133.2 (C-1), 133.9 (C-3'), 134.7 (C-5a), 142.8 (C-3a), 143.6 (C-7'a), 148.1 (C-7), 166.6 (CO_2_H), 169.2 (C-2'), 177.3 (C-2), 178.8 (C-9). HMRS ((–)-ESI): *m/z* = 434.05486 (calcd. 434.05492 for C_22_H_13_Cl^35^N_3_O_5_, [M−H]^−^); *m/z* = 436.05163 (calcd. 436.05295 for C_22_H_13_Cl^37^N_3_O_5_, [M+2−H]^−^). IR: ν 3414, 3150, 3056, 2917, 2851, 1691, 1613, 1530, 1451 cm^−1^. *Anal*. Calcd. for C_22_H_14_ClN_3_O_5_ (435.81): C, 60.63; H, 3.24; N, 9.64. Found C, 60.38; H, 3.16; N, 9.48.

*(E)-1-(6'-Chloro-2'-oxoindolin-3'-ylidene)-6-ethyl-2,3,6,9-tetrahydro-2,9-dioxo-1H-pyrrolo[3,2-f]quinoline-8-carboxylic acid* (**3c**): Yield: 0.38 g (88%); m.p. > 300 °C. ^1^H-NMR (DMSO-*d*_6_): δ = 1.46 (t, *J* = 7.0 Hz, CH_3_CH_2_, 3H), 4.62 (q, *J* = 7.0 Hz, 2H, CH_2_Me), 6.84 (s, 1H, 7'-H), 7.09 (d, *J* = 8.4 Hz, 1H, 5'-H), 7.39 (d, *J* = 9.0 Hz, 1H, 4-H), 8.02 (d, *J* = 9.0 Hz, 1H, 5-H), 8.77 (d, *J* = 8.4 Hz, 1H, 4'-H), 8.94 (s, 1H, 7-H), 10.74 (s, 1H, N(1')-H/exchangeable with D_2_O), 11.05 (s, 1H, N(3)-H/exchangeable with D_2_O), 15.31 (s, 1H, CO_2_H/exchangeable with D_2_O). ^13^C-NMR (DMSO-*d*_6_): δ = 15.6 (CH_3_CH_2_), 50.1 (CH_2_Me), 108.8 (C-8), 110.3 (C-7'), 116.8 (C-4), 117.8 (C-9b), 120.7 (C-3'a), 121.7 (C-5'), 123.1 (C-5), 124.8 (C-9a),130.0 (C-1), 131.8 (C-4'), 134.6 (C-5a), 136.4 (C-7'a), 138.1 (C-6'), 145.0 (C-3a), 146.8 (C-3'), 147.2 (C-7), 166.9 (CO_2_H), 168.1 (C-2'), 169.9 (C-2), 177.5 (C-9). ^1^HMRS ((+)-ESI): *m/z* = 436.06922 (calcd. 436.06947 for C_22_H_15_ Cl^35^N_3_O_5_, [M+H]^+^); *m/z* = 438.06726 (calcd. 438.06751 for C_22_H_15_Cl^37^N_3_O_5_, [M+2+H]^+^). IR: ν 3412, 3193, 1696, 1610, 1447 cm^−1^. *Anal*. Calcd. for C_22_H_14_ClN_3_O_5_ (435.82): C, 60.63; H, 3.24; N, 9.64. Found C, 60.42; H, 3.12; N, 9.51.

*(E)-1-(5'-Bromo-2'-oxoindolin-3'-ylidene)-6-ethyl-2,3,6,9-tetrahydro-2,9-dioxo-1H-pyrrolo[3,2-f]quinoline-8-carboxylic acid* (**3d**): Yield: 0.36 g (76%); m.p. > 300 °C. ^1^H-NMR (DMSO-*d*_6_): δ = 1.45 (t, *J* = 7.0 Hz, 3H, CH_3_CH_2_), 4.60 (q, *J* = 7.0 Hz, 2H, CH_2_Me), 6.79 (d, *J* = 8.3 Hz, 1H, 7'-H), 7.40 (d, *J* = 9.0 Hz, 1H, 4-H), 7.55 (d, *J* = 8.3 Hz, 1H, 6'-H), 8.03 (d, *J* = 9.0 Hz, 1H, 5-H), 8.95 (s, 1H, 7-H), 8.97 (s, 1H, 4'-H), 10.55 (s, 1H, N(1')-H/exchangeable with D_2_O), 11.09 (s, 1H, N(3)-H/exchangeable with D_2_O), 15.30 (s, 1H, CO_2_H/exchangeable with D_2_O). ^13^C-NMR (DMSO-*d*_6_): δ = 15.5 (CH_3_CH_2_), 50.1 (CH_2_Me), 108.9 (C-8), 112.2 (C-7'), 116.8 (C-4), 117.8 (C-9b), 123.4 (C-5), 123.5 (C-5'), 123.7 (C-3'a), 125.0 (C-9a), 130.9 (C-1), 132.3 (C-4'), 134.6 (C-5a), 136.1 (C-6'), 136.2 (C-3'), 144.7 (C-7'a), 145.3 (C-3a), 147.2 (C-7), 166.8 (CO_2_H), 167.7 (C-2'), 170.0 (C-2), 177.6 (C-9). HMRS ((–)-ESI): *m/z* = 478.00430 (calcd. 478.00441 for C_22_H_13_Br^79^N_3_O_5_, [M−H]^−^); *m/z* = 480.00231 (calcd. 480.00267 for C_22_H_13_ Br^81^N_3_O_5_, [M+2−H]^−^). IR: ν 3417, 3152, 3065, 1694, 1610, 1607 cm^−1^. *Anal*. Calcd. for C_22_H_14_BrN_3_O_5_ (480.27): C, 55.02; H, 2.94; N, 8.75. Found C, 54.83; H, 2.85; N, 8.66.

*(E)-1-(6'-Bromo-2'-oxoindolin-3'-ylidene)-6-ethyl-2,3,6,9-tetrahydro-2,9-dioxo-1H-pyrrolo[3,2-f]quinoline-8-carboxylic acid* (**3e**): Yield: 0.44 g (92%); m.p. > 300 °C. ^1^H-NMR (DMSO-*d*_6_): δ = 1.40 (t, *J* = 6.9 Hz, 3H, CH_3_CH_2_), 4.56 (q, *J* = 6.9 Hz, 2H, CH_2_Me), 6.99 (s, 1H, 7'-H), 7.23 (d, *J* = 8.6 Hz, 1H, 5'-H), 7.38 (d, *J* = 9.0 Hz, 1H, 4-H), 8.01 (d, *J* = 9.0 Hz, 1H, 5-H), 8.68 (d, *J* = 8.6 Hz, 1H, 4'-H), 8.90 (s, 1H, 7-H), 10.72 (s, 1H, N(1')-H/exchangeable with D_2_O), 11.05 (s, 1H, N(3)-H/exchangeable with D_2_O), 15.30 (s, 1H, CO_2_H/exchangeable with D_2_O). ^13^C-NMR (DMSO-*d*_6_): δ = 15.6 (CH_3_CH_2_), 50.1 (CH_2_Me), 108.8 (C-8), 113.1 (C-7'), 116.8 (C-4), 117.8 (C-9b), 121.0 (C-3'a), 123.1 (C-5), 124.4 (C-5'), 124.7 (C-9a), 127.1 (C-6'), 130.1 (C-1), 131.8 (C-4'), 134.6 (C-5a), 136.5 (C-3'), 145.0 (C-3a), 146.8 (C-7'a), 147.2 (C-7), 166.8 (CO_2_H), 167.9 (C-2'), 170.0 (C-2), 177.5 (C-9). HMRS ((+)-ESI): *m/z* = 480.01908 (calcd. 480.01896 for C_22_H_15_Br^79^N_3_O_5_, [M+H]^+^); *m/z* = 482.01698 (calcd. 482.01723 for C_22_H_15_ Br^81^N_3_O_5_, [M+2+H]^+^). IR: ν 3412, 1696, 1609, 1447 cm^−1^. *Anal*. Calcd. for C_22_H_14_BrN_3_O_5_ (480.27): C, 55.02; H, 2.94; N, 8.75. Found C, 55.13; H, 3.02; N, 8.59.

*(E)-6-Ethyl-1-(5'-nitro-2'-oxoindolin-3'-ylidene)-2,9-dioxo-2,3,6,9-tetrahydro-1H-pyrrolo[3,2-f]quinoline-8-carboxylic acid* (**3f**): Yield: 0.39 g (87%); m.p. > 300 °C.^1^H-NMR (DMSO-*d*_6_): δ = 1.46 (t, *J* = 7.1 Hz, 3H, CH_3_CH_2_), 4.62 (q, *J* = 7.1, 2H, CH_2_Me), 7.02 (d, *J* = 8.6 Hz, 1H, 7'-H), 7.40 (d, *J* = 8.8 Hz, 1H, 4-H), 8.07 (d, *J* = 8.8 Hz, 1H, 5-H), 8.30 (d, *J* = 8.6 Hz, 1H, 6'-H), 8.95 (s, 1H, 7-H), 9.72 (s, 1H, 4'-H), 11.16 (s, 1H, N(3)-H/exchangeable with D_2_O), 11.34 (s, 1H, N(1')-H/exchangeable with D_2_O), 15.23 (s, 1H, CO_2_H/exchangeable with D_2_O). ^13^C-NMR (DMSO-*d*_6_): δ = 15.6 (CH_3_CH_2_), 50.2 (CH_2_Me), 109.0 (C-8), 110.4 (C-7'), 117.0 (C-4), 117.3 (C-9b), 121.6 (C-3'a), 124.2 (C-5), 124.9 (C-9a), 125.5 (C-4'), 129.6 (C-6'), 132.4 (C-1), 134.7 (C-5a), 134.8 (C-3'), 142.3 (C-5'), 145.9 (C-3a), 147.4 (C-7), 150.5 (C-7'a), 166.7 (CO_2_H), 168.2 (C-2'), 170.1 (C-2), 177.4 (C-9). HMRS ((+)-ESI): *m/z* = 447.09357 (calcd. 447.09353 for C_22_H_15_N_4_O_7_, [M+H]^+^); *m/z* = 469.07530 (calcd. 469.07547 for C_22_H_14_N_4_O_7_Na), [M+Na]^+^. IR: ν 3413, 3187, 1696, 1612, 1581, 1519, 1451 cm^−1^. *Anal*. Calcd. for C_22_H_14_N_4_O_7_ (446.37): C, 59.20; H, 3.16; N, 12.55. Found C, 59.03; H, 3.12; N, 12.44.

### 3.5. Cell Culture Conditions

The human chronic myelogenous leukemia K562 (ATCC^®^ CCL-243^TM^) and human acute monocytic leukemia THP-1 (ATCC^®^ TIB-202^TM^) suspension cells were maintained in RPMI-1640, while adherent HepG2 (human hepatocellular carcinoma, ATCC^®^ HB-8065^TM^), MCF7 (human breast adenocarcinoma, ATCC^®^ HTB-22^TM^) were cultured in DMEM medium. Adherent Caco-2 (human colorectal adenocarcinoma, ATCC^®^ HTB-37^TM^), HEK-293 (human embryonic epithelial cells, ATCC^®^ CRL-1573^TM^) and L-929 (mouse subcutaneous connective tissue fibroblast, ATCC^®^ CCL-1^TM^) cells were cultured in MEM medium. The media were supplemented with 10% (v/v) heat inactivated FBS, penicillin G (100 U/mL) and streptomycin (100 mg/mL), and cells were incubated at 37 °C in a 5% CO_2_ humidified incubator. The media were changed every 2–3 days and subcultured when the cell population density reached to 70%–80% confluence. Cells were seeded at an appropriate density according to each experimental design.

### 3.6. Cell Viability (Antiproliferative) Assay

The antiproliferative activity of the synthesized compounds was assessed using the MTT cell viability assay [18]. Briefly, 2 × 10^4^ of the non-adherent K562 or THP-1 cells, in a 100 μL of RPMI-1640 medium, or 7 ° 10^3^ of adherent HepG2, MCF-7, Caco-2, HEK-293 or L-929, in a same volume of the proper media, were seeded in each well of a 96 well-plate. After 24 h, the media was removed by aspiration and replaced by fresh media containing the test compound at varying concentrations (0.0 to 40.0 µM, for cancerous cells) or (0.0 to 80.0 µM, for noncancerous cells), and incubated for the desired time in a 5% CO_2_-cell culture incubator. Alternatively, cultured K562 cells were incubated with 12.0 µM of the test compound for different time-points (0, 2, 4, 8, 12, 24, 48 and 72 h) before analysis. At the end of incubation period with the different molecules, media were removed by aspiration and the cells gently rinsed with PBS to remove residual compound. 100 μL of MTT (0.5 mg/mL in PBS) were added to each well and incubated for 4 h at 37 °C. The MTT solution was removed gently by aspiration and the formazan crystals dissolved in 100 μL of DMSO. Absorbance was measured within 60 min at 595 nm using an E Max Precision Microplate reader (Molecular Devices, Sunnyvale, CA, USA). Cell viability at a given concentration was determined from the following expression:

Percentage Viability = (A_test compound_ − A_blank_/A_control_ − A_blank_) × 100 (1)

where A_test compound_ = absorbance of wells with cells exposed to test compound in media; A_control_ = absorbance of wells with cells in media and A_blank_ = absorbance of wells with DMSO only, without cells. Each concentration was tested in triplicates in each of three independent experiments, using two different stock solutions, and on cells of different passage numbers. Since the maximum solubility of the synthesized molecules was 50 µM in warmed PBS, stock solutions of 1,000-fold and 2,000-fold of the different final concentrations of test compounds were prepared in 1:1 DMSO: PBS or 1:2 DMSO: PBS. Therefore, in all experiments, the final concentrations of DMSO were 0.025 or 0.05%. Similar concentrations of DMSO were added to the control of untreated cells, and showed no effect on cell viability, when compared to the viability of cells without DMSO.

The concentration of test compound leading to 50% inhibition of viability (IC_50_), compared to untreated cells, was determined from the sigmoidal curve obtained by plotting the percentages of viability *versus* logarithmic concentration of test compound using non-linear regression analysis of GraphPad Prism 6 software (San Diego, CA, USA). Under similar conditions, K562 cells were treated with 4.0, 8.0 or 12.0 µM etoposide as a positive control for the MTT assay.

### 3.7. Analysis of Apoptosis

The percentage of K562 cells undergoing apoptosis was determined by using the Muse^TM^ Annexin V & Dead Cell Assay (EMD Millipore Bioscience, Darmstadt, Germany) which utilizes a fluorescent dye conjugated to Annexin V to detect phosphatidylserine (PS) on the external membrane of apoptotic cells [35]. A dead cell marker (7-AAD; 7-aminoactinomycin D) is also used as an indicator of cell membrane structural integrity in the same kit [36]. It is excluded from live, healthy cells, as well as early apoptotic cells. Four populations of cells can be distinguished in this assay when analyzed by the flow cytometer-based instrument Muse^TM^ Cell Analyzer: (1) the non-apoptotic cells: Annexin V^−^ and 7-AAD^−^, (2) the early apoptotic cells: Annexin V^+^ and 7-AAD^−^, (3) the late stage apoptotic and dead cells: Annexin V^+^ and 7-AAD^+^ and 4) the mostly nuclear debris (necrosis): Annexin V^−^ and 7-AAD^+^. The treated K562 cells with different concentrations of compound **3b** or **3d** for 24 h were incubated with the Annexin V & Dead Cell fluorescent dyes and incubated in dark place for 20 min before analysis. All samples were kept in ice and analyzed by the flow cytometer within 1 h. Data of apoptosis induction by compound **3b** and **3d** were calculated from three independent experiments.

### 3.8. Statistical Analysis

Data presented are the means ± S.D. of results from a minimum of three independent experiments with similar patterns. Statistical analysis was performed using one-way ANOVA and Student’s *t*-test. A *p* < 0.05 value was considered statistically significant.

## 4. Conclusions

In this study, we have synthesized some specific pyridone-annelated isoindigos and assessed their antiproliferative activities towards different hematological and solid tumor cell lines. Our results showed that two of the synthesized isoindigos having chlorine and bromine atoms at the 5'**-**position of the benzo-ring E display strong antiproliferative activities towards all the tested cancer cell lines, but not noncancerous cells, suggesting their potential use as anticancer agents. Our results suggest that the antiproliferative effect of both compounds **3b** and **3d** in K562 cells is mediated by apoptosis. Therefore, it will be an exciting challenge to further characterize the pharmacology of these active compounds and, in particular, their anticancer pharmacological actions.
